# Comparing the cost-effectiveness of the Otago Exercise Programme among older women and men: A secondary analysis of a randomized controlled trial

**DOI:** 10.1371/journal.pone.0267247

**Published:** 2022-04-20

**Authors:** Jennifer C. Davis, Chun Liang Hsu, Cindy Barha, Deborah A. Jehu, Patrick Chan, Cheyenne Ghag, Patrizio Jacova, Cassandra Adjetey, Larry Dian, Naaz Parmar, Kenneth Madden, Teresa Liu-Ambrose

**Affiliations:** 1 Aging, Mobility, and Cognitive Neuroscience Laboratory, Department of Physical Therapy, University of British Columbia, Vancouver, British Columbia, Canada; 2 Djavad Mowafaghian Centre for Brain Health, Vancouver Coastal Health Research Institute, Vancouver, British Columbia, Canada; 3 Centre for Hip Health and Mobility, Vancouver Coastal Health Research Institute, Vancouver, British Columbia, Canada; 4 Social & Economic Change Laboratory, Faculty of Management, University of British Columbia, Kelowna, British Columbia, Canada; 5 Hinda and Arthur Marcus Institute for Aging Research, Hebrew SeniorLife, Roslindale, Massachusetts, United States of America; 6 Interdisciplinary Health Sciences Department, Augusta University, Augusta, Georgia, United States of America; 7 Division of Geriatric Medicine, Department of Medicine, Faculty of Medicine, University of British Columbia, Vancouver, British Columbia, Canada; Cardiff University, UNITED KINGDOM

## Abstract

**Objective:**

Using stratified analyses, we examined the cost-effectiveness of the Otago Exercise Programme (OEP), from a health care system perspective, among older women and men who have previously fallen.

**Methods:**

This study was a secondary stratified analysis (by women and men), of a 12-month prospective economic evaluation of a randomized clinical trial (OEP compared with usual care). Three hundred and forty four community-dwelling older adults (≥70; 172 OEP (110 women; 62 men), 172 usual care (119 women; 53 men)) who sustained a fall in the past 12 months and received a baseline assessment at the Vancouver Falls Prevention Clinic, Canada were included. A gender by OEP/usual care interaction was examined for the falls incidence rate ratio (IRR). Outcome measures stratified by gender included: falls IRR, incremental cost-per fall prevented (ICER), incremental cost per quality adjusted life year (QALY, ICUR) gained, and mean total health care resource utilization costs.

**Results:**

Men were frailer than women at baseline. Men incurred higher mean total healthcare costs $6794 (SD: $11906)). There was no significant gender by OEP/usual care interaction on falls IRR. The efficacy of the OEP did not vary by gender. The adjusted IRR for the OEP group demonstrated a 39% (IRR: 0.61, CI: 0.40–0.93) significant reduction in falls among men but not women (32% reduction (IRR: 0.69, CI: 0.47–1.02)). The ICER showed the OEP was effective in preventing falls and less costly for men, while it was costlier for women by $42. The ICUR showed the OEP did not impact quality of life.

**Conclusion:**

Future studies should explore gender factors (i.e., health seeking behaviours, gender related frailty) that may explain observed variation in the cost-effectiveness of the OEP as a secondary falls prevention strategy.

**Trial registrations:**

ClinicalTrials.gov Protocol Registration System

Identifier: NCT01029171; URL: https://clinicaltrials.gov/ct2/show/NCT01029171

Identifier: NCT00323596; URL: https://clinicaltrials.gov/ct2/show/NCT00323596

## Introduction

The costs of falls incurred by older adults are substantial [1]. Annual medical costs attributable to nonfatal and fatal falls were estimated at $50.0 billion (2015 US prices) [2]. Up to 20% of falls result in serious injury that may require extended medical care or rehabilitation services [3]; these exacerbate the economic burden that falls impose on the health care system.

Emerging evidence suggests that costs sustained by older adults differ between women and men. Among older adults who sustained a prior fall, men were more likely to visit an allied health professional post-fall compared with women [4]. Women were more likely to visit a specialist consultant or have an outpatient visit provided after a fall compared with men. The overall fall-related healthcare system costs for women, who fall more often, were almost three times greater than men [5, 6]. At a societial level, women accounted for the larger total fall-related costs than men. However, at an individual level, men aged 65–69 incurred greater mean fall-related cost, and also demonstrated the greatest mortality risk upon admission [5, 7].

Fall rates and risk of fall-related injury also differ between men and women. As adults age, the rate of injurious falls are higher in community dwelling women [8]; women demonstrate longer post-injury survival compared with men [9]. Fall-related hospitalization rates are higher among women (30 per 1000) compared with men (20 per 1000) and advance with age. For example, women aged 90 years and older have a reported fall-hospitalization rate of 69 per 1000 compared with 6 per 1000 among women aged 65–69 years. Similar trends, albeit lower falls-related hospitalization rates are observed for men [10].

The Otago Exercise Programme (OEP), has proven effective for primary [11–13] and secondary prevention [14] of falls among community-dwelling adults aged 65 years and older. Evidence supporting the OEP [15] include the Centers for Disease Control and Prevention Compendium of Effective Fall Prevention Interventions [16], the National Institute for Health and Care Excellence [17], systematic reviews (Cochrane) [18] and high quality clinical trials [13, 14]. In New Zealand, the OEP effectively reduced falls rates by up to 46% among older adults [12]. However, this study did not present results stratified by gender or examine a gender by OEP interaction on falls rates [12].

The evidence for the efficacy of exercise interventions for the secondary prevention of falls comparing women and men is unequivocal. One meta-analysis of four studies demonstrated that the OEP was efficacious for the primary prevention of falls for women and men (eg. Women: 26% reduction, incidence rate ratio (IRR): 0.74 (0.63–0.88) and Men: 60% reduction, IRR: 0.40 (0.22–0.74) [19]. The efficacy of a physical activity intervention that included structured physical activity and health eduction in reducing rates of serious fall injuries was moderated by gender (for men: rate ratio 0.54: [0.31 to 0.95] and for women: 1.07 [0.75 to 1.53]; p = 0.043 for interaction). This moderation effect of gender was also demonstrated for fall related hospital admissions (for men: 0.41 [0.19 to 0.89] and for women; 1.10 [0.65 to 1.88]; p = 0.039 for interaction) [20]. However, these findings are hypothesis generating, because neither study was powered to examine differences among women and men apriori.

The OEP is a cost-effective primary and cost-effective secondary prevention strategy [12, 13, 21] [21]. However, whether the OEP is cost-effective for both women and men is unknown. This is important to determine to guide future resource allocation and implementation decisions. Our primary objective was to examine whether the OEP is cost-effective for women and men. We conducted a stratified analysis (women and men) to determine the ICERs–the incremental cost per fall prevented and the incremental cost per Quality Adjusted Life Year (QALY, ICUR) gained/lost of the OEP plus usual care compared with usual care alone. We used a 12-month time horizon aligning with the OEP intervention and a Canadian health care system perspective.

## Materials and methods

This is a secondary analysis of a randomized clinical trial with a concurrent economic evaluation that examined the cost-effectiveness and cost-utility of the OEP, stratified by women and men. The study protocol, principal randomized clinical trial findings and primary economic evaluation findings are published [14, 21, 22].

### Overview

The economic evaluation was based on 344 adults (172 OEP; 172 usual care) aged 70 years and older who sustained a fall in the previous 12 months who participated in a 12-month parallel, single-blinded, randomized clinical trial of the OEP [14]. This trial took place in the Vancouver area of British Columbia, Canada. Participants were recruited from the Falls Prevention Clinic at Vancouver General Hospital (www.fallsclinic.com).

Two registrations for this trial [14] include the: 1) proof-of-concept study [23] and 2) the original registration (ClinicalTrials.gov identifier: NCT00323596 and NCT01029171).

Ethical approval was obtained from the Vancouver Coastal Health Research Institute (V10-70171, May 11, 2004) and the University of British Columbia’s Clinical Research Ethics Board (H04-70171, May 11, 2004). All participants provided written and informed consent.

### Inclusion criteria

Participants were included if they: 1) were aged ≥ 70 years; 2) were referred by a medical professional to the Falls Prevention Clinic and sustained a non-syncopal low trauma fall in the previous 12 months; 3) were able to understand, speak, and read English proficiently; 4) had an Mini-Mental State Examination (MMSE) [24] score ≥ 24/30; 5) had a Physiological Profile Assessment (PPA) ^©^ [25] score of at least 1.0 SD above age-normative value OR Timed Up and Go Test (TUG) [26] performance of greater than 15 seconds OR one additional non-syncopal fall in the previous 12 months; 6) were expected to live greater than12 months (based on the geriatricians’ expert opinion); 7) were living in the Vancouver area; 8) were community-dwelling; 9) were able to walk 3 meters with or without an assistive device; and 10) were able to provide written informed consent.

### Exclusion criteria

Participants were excluded if they: 1) were previously diagnosed with or suspected (by the geriatrician) to have neurodegenerative disease or dementia; 2) had a stroke; or 3) had a history indicative of carotid sinus sensitivity (i.e., syncopal falls).

### Measures

Assessors were blinded to the participants’ assignments [27]. Stratified by gender, we reported baseline demographic variables that included: age (years), education, comorbidities (using the Functional Comorbidity Index [28] where a score range 0–18, 0 = best, indicating no comorbid illness), gait speed (meters per second), depression (using the 15-item Geriatric Depression Scale [29, 30]; range 0–15, 0 = best; scores ≤ 5 are normal), the ability to live independently (using the Lawton and Brody [31] Instrumental Activities of Daily Living Scale; range 0–8, 8 = best), and global cognitive function (using the MMSE [32] and the Montreal Cognitive Assessment (MoCA) [33]; range 0–30 points for each measure, higher = better). Other descriptive measures, included fall risk assessed by the Physiological Profile Assessment (PPA) [25] (observed range -2 to 4, lower = better; 0–1 indicates mild risk, 1–2 indicates moderate risk, 2–3 indicates high risk, and 3 and above indicates marked risk).General balance and mobility was assessed using the Short Physical Performance Battery (SPPB) [34] and the Timed Up and Go (TUG) test [35]. The SPPB has a range of 0–12, with 12 indicating the best performance. An SPPB score of ≤ 9/12 predicts subsequent disability [34]. A TUG score of ≥ 13.5 (range of 0–84) seconds indicates high fall risk; lower scores on the TUG indicate lower fall risk [32]. We used monthly fall diary calendars to track all falls for each participant during the 12-month study period.

### Intervention

Participants received a 12-month intervention–the OEP–an individualized home-based balance and strength retraining program [11]. A licensed physical therapist visited the home of participants and prescribed exercises from a manual. A total of 6 visits during the first 6 months were provided. Intervention details are described in further detail in the primary paper [14].

### Usual care

Participants randomized to the control group received usual care that included a baseline assessment at the Falls Prevention Clinic with followup as requested by the geriatrician.

### Overview of economic evaluation

The outcome of our gender-stratified cost effectiveness analysis is the incremental ICER (i.e., ICER = Δ Cost/Δ Effectiveness) [36]. Effectiveness was measured by: 1) the difference in the number of falls prevention between the OEP and the usual care group and 2) the difference in the mean QALYs between the OEP and the usual care group. The economic evaluation utilized a 12-month time horizon and a Canadian health care system perspective. All statistical analyses were carried out using STATA version 13.0.

### Costs

#### Costs of delivering the OEP

Costs of delivering the OEP were estimated to reflect real-world costs of delivering the OEP in the community. Costs included physiotherapist time with participants to deliver the intervention, training time to train physiotherapists to deliver the OEP and cost of weights included for the OEP.

#### Health resource utilization costs

We used a self-report questionnaire and monthly cost-diaries to track total healthcare resource utilization prospectively over 12 months [37]. Health resource utilization data were not imputed due to the high percentage of complete data obtained [38]. Unit costs for healthcare cost items were previously reported [21]. A unit cost from the British Columbia Medical Serviced Plan (https://www2.gov.bc.ca/gov/content/health/practitioner-professional-resources/msp/physicians/payment-schedules/msc-payment-schedule) 2018 fee for service price list was assigned for each component of HRU. Hospital admission costs were based on a fully allocated cost model of a tertiary care hospital (a hospital that delivers a higher level of specialized care). A fully allocated cost model utilized a micro-costing approach whereby a researcher identified all hospital resources utilized by individual patients in specific departments (i.e., Emergency Department) to depict averages costs by time spent in a particular ward. Unit costs for specialized services (e.g., physiotherapy) were taken from the relevant British Columbia Association website. All costs were reported in 2019 Canadian Dollars. Discounting was not applied due to the 12-month time horizon. The total health care related costs of delivering the OEP for 12 months were compared with usual care.

#### Effects: Falls and quality adjusted life years

We used monthly fall diary calendars to track all falls for each participant during the 12-month study period. The number of falls per person year was calculated as the sum of all falls experienced divided by the cumulative exposure time (in years) across participants. In this study, exposure time refers to the time participants were followed in the study (i.e., baseline to final assessment or withdrawal date). Falls data were not imputed due to the high percentage of complete data obtained. Between-group differences in rate of falls were modeled using negative binomial regression. Model fit was assessed by visualizing the falls count variable to examine whether its distribution followed a negative binomial distribution. First, a gender by group interaction was examined. Second, the negative binomial regression model was controlled for age and exposure time, and stratified by gender.

Quality adjusted life years were assessed using the Short-Form 6D (SF-6D) at baseline, 6 and 12-months [39]. The SF-6D captures physical functioning, role limitations, social functions mental health, bodily pain and vitality. In addition, the SF-6D describes 18,000 discrete health states and will likely capture small changes in health status. Quality adjusted life years were estimated to account for the quality of life of a patient (measured using health utilities assessed with the SF-6D) in a given health state and the time spent in that health state [40]. To calculate QALYs, we utilized area under the curve analysis using the trapezoid method. We used linear regression to adjust for imbalances in baseline utilities between OEP and ‘usual care’. Variables included in the regression model were baseline utility and group. An incremental QALY was estimated by calculating the difference in mean adjusted QALY of the OEP intervention group compared with the mean adjusted QALY of the usual care group.

#### Statistical analysis of cost effectiveness and cost utility analyses

We calculated the incremental cost effectiveness ratio (ICER) as the incremental cost per number of falls prevented of the OEP versus usual care alone (comparator). We followed recommendations by Oostenbrink, Briggs, Manca and colleagues for multiple imputation of missing cost and effectiveness data.[41–44] For all discrete time points, we used a combination of multiple imputation and bootstrapping to estimate uncertainty caused by missing values [43, 44]. We used nonparametric bootstrapping nested in multiple imputation to model uncertainty around the estimates for costs and effectiveness. For each of the five cycles, we imputed missing values and bootstrapped the complete dataset. For each cycle of imputation and bootstrapping, we calculated the total healthcare resource use cost, fall related resource use cost and number of falls per participant by group allocation. We averaged results of each cycle of imputation for participants in the two groups (OEP versus usual care). We evaluated the contribution of each cost item in relation to the total healthcare resource use estimated for each group. We used Fiellers’ method to generate 95% confidence ellipses for the joint distribution of cost and effectiveness outcomes [45].

*Unique ICURs were estimated from QALYs*. We calculated the incremental cost per QALY for the OEP plus usual care versus usual care alone. We used 5000 bootstrapped replications of mean cost difference and mean QALY differences [46].

## Results

Baseline study characteristics stratified by gender are presented in Table 1. The OEP group consisted of 110 women and 62 men. The usual care group included 119 women and 53 men. In the OEP group, 26 individuals were lost to followup (20 women; 6 men). In the usual care group, 22 individuals were lost to followup (13 women; 9 men).

**Table 1:** Characteristics of randomized clinical trial participants stratified by gender.

	Women n = 229	Men n = 115	OEP Women n = 110	Usual care Women n = 119	OEP Men n = 62	Usual care Men n = 53
Variables at baseline	Mean (SD) or n (%) or Median (IQR)	Mean (SD) or n (%) or Median (IQR)	Mean (SD) or n (%) or Median (IQR)	Mean (SD) or n (%) or Median (IQR)	Mean (SD) n (%) or Median (IQR)	Mean (SD) n (%) or Median (IQR)
*Descriptive variables*						
Age, years	81.2 (6.0)	82.2 (6.2)	81.2 (6.4)	81.3 (5.8)	81.2 (3.8)	83.2 (6.6)
Education, ≥ high school	210 (92%)	104 (90%)	101 (92%)	109 (92%)	55 (89%)	49 (92%)
FCI	4.2 (2.2)	3.7 (1.9)	4.2 (2.3)	4.1 (2.1)	3.8 (2.0)	3.7 (1.6)
MMSE	28.0 (1.6)	27.3 (1.7)	27.8 (1.6)	28.2 (1.5)	27.4 (1.6)	27.2 (1.8)
MoCA	23.3 (3.4)	22.8 (3.2)	23.0 (3.4)	23.6 (3.4)	22.8 (3.5)	22.8 (3.0)
SPPB	8.0 (2.2)	7.5 (2.3)	7.9 (2.1)	8.2 (2.3)	7.9 (2.4)	7.1 (2.2)
TUG (secs)	16.2 (6.5)	17.5 (7.0)	16.3 (7.1)	16.1 (6.0)	16.5 (7.0)	18.6 (7.1)
PPA	1.9 (1.1)	2.0 (1.1)	2.0 (1.0)	1.8 (1.2)	1.8 (1.2)	2.1 (1.0)
*Primary Clinical and Economic Outcome Variables*						
Mean number of falls (n)	1.4 (2.9)	2.4 (3.3)	1.1 (1.5)	1.7 (3.7)	1.9 (2.4)	3.0 (4.0)
Total Exposure, days, mean (SD)	336.3 (84.1)	342.1 (75.1)	328.5 (93.6)	343.5 (75.0)	342.7 (72.0)	341.4 (79.4)
SF-6D	0.718 (0.079)	0.725 (0.078)	0.713 (0.085)	0.722 (0.073)	0.723 (0.081)	0.728 (0.076)
*Secondary Outcome Variables*						
Gait speed, m/s	0.8 (0.2)	0.8 (0.2)	0.8 (0.2)	0.9 (0.2)	0.8 (0.2)	0.8 (0.2)
GDS	2.8 (2.5)	3.0 (2.6)	2.7 (2.4)	2.9 (2.6)	3.0 (2.4)	3.0 (2.8)

FCI: Functional Comorbidity Index; MMSE: Mini-Mental State Examination (range: 0 to 30); MoCA: Montreal Cognitive Assessment (range: 0 to 30); SPPB: Short Physical Performance Battery (range: 0 (worst) to 12 (best)); TUG: Time-up-and-go (range: 1 (normal) -5 (abnormal function)); PPA: Physiological Profile Assessment (range: -2 to 3); SF-6D: Short Form-6Domain (range: 0.291–1.0); GDS: Geriatric Depression Scale (0-normal to 15-severe depression).

### Healthcare use and costs

Our gender-stratified analyses indicate the mean total healthcare costs were greater for men (OEP: $5690 CAD (SD: $6754); usual care: $6794 CAD (SD: $11906)) compared with women (OEP: $3691 CAD (SD: $3353); usual care: $3665 CAD (SD: $5026)) regardless of whether participants were randomized to the OEP group or the usual care group (see Table 2).

**Table 2:** Economic evaluation results of the cost-effectiveness and cost-utility study (complete case analyses) stratified by gender.

	OEP Mean (SD) Women	Usual Care Mean (SD) Women	OEP Mean (SD) Men	Usual care Mean (SD) Men
**Costs**				
Cost of delivering OEP per person (2019 CAD $)	393 (45)	0 (usual care)	393 (45)	0 (usual care)
Mean total healthcare resource use cost^Ý^ (2019 CAD $) per person *Incremental cost*	3691 (3353)	3665 (5026)	5690 (6754)	6794 (11906)
	26 (569)	reference^||^	-1104 (1773)	reference^||^
**Falls**				
Number of falls	1.1 (1.4)	1.7 (3.7)	1.9 (2.4)	3.0 (4.0)
*Incremental number of falls*	-0.6 (0.4)	reference^||^	-1.2 (0.6)	reference^||^
**Cost-effectiveness analysis** ^Ý^				
*Incremental cost per fall prevented*	-42	reference^||^	dominates	reference^||^
**QALYs**				
*Unadjusted QALY (SF-6D)*	0.791 (0.078)	0.783 (0.062)	0.804 (0.065)	0.803 (0.065)
*Adjusted QALY* ^Ý^				
SF-6D	0.787 (0.0670	0.787 (0.058)	0.798 (0.056)	0.795 (0.053)
*Adjusted Incremental QALY* ^ý^	0.001 (0.008)	reference^||^	0.005 (0.011)	reference^||^
**Cost-utility analysis** ^Ý^				
*Incremental cost per QALY*				
Based on SF-6D	28 582 (95% CI: -42556 to 99721) Upper right quadrant	reference^||^	238913 (95% CI: -230270 to 247557) Lower left quadrant	reference^||^

Notes: The time horizon for this analysis was 12-months.

* p<0.05

Ý ICER based on total health resource utilization costs and cost of delivering programs

ý Incremental QALYs are adjusted for the baseline utility using a linear regression model

|| Reference indicates that the comparator is the usual care (control) group.

### Adverse events and mortality

Three deaths occurred in the OEP group (1 female and 2 men) and one death occurred in the usual care group (1 female); none of these were attributable to the intervention.

#### Cost effectiveness analysis–Complete case analysis

The interaction between gender and group was not statistically significant for predicting falls IRR. Compared with usual care, the adjusted IRR for the OEP group demonstrated a 39% (IRR: 0.61, CI: 0.40–0.93) significant reduction in falls among men. The adjusted IRR for the OEP group demonstrated a 31% (IRR: 0.69, CI: 0.47–1.02) non-significant reduction in falls among women.

Based on the point-estimates of our base case cost effectiveness analysis among men, the OEP resulted in lower incremental healthcare costs and was more efficacious (i.e., resulted in a significant reduction in the falls IRR) than usual care. Based on the point-estimates of our base case cost effectiveness analysis among women, the OEP resulted in higher incremental healthcare costs and was not more efficacious (i.e., non-significant reduction in the IRR for falls) than usual care.

#### Cost utility analysis–complete case and imputed case analysis

Table 2 details the mean QALYs for the complete case analyses. Table 3 details the imputed data set.

**Table 3:** Economic evaluation results of the cost-effectiveness and cost-utility study (imputed case analyses) stratified by gender.

	OEP Mean (SD) Women	Usual Care Mean (SD) Women	OEP Mean (SD) Men	Usual care Mean (SD) Men
**Costs**				
Cost of delivering OEP per person (2019 CAD $)	393 (45)	0 (usual care usual care)	393 (45)	0 (usual care)
Mean healthcare resource use cost^Ý^ (2019 CAD $) per person *Incremental cost*	4018 (4936)	4018 (4936)	5690 (6700)	6794 (11795)
	26 (79)	reference^||^	-1104 (246)	reference^||^
**Falls**				
Mean number of falls	1.1 (1.5)	1.7 (3.7)	1.9 (2.4)	3.0 (3.9)
*Incremental mean number of falls*	-0.6 (0.1)	reference^||^	-1.2 (0.1)	reference^||^
**Cost-effectiveness analysis** ^Ý^				
*Incremental cost per fall prevented*	-42 (CI: -293 to 207)	reference^||^	dominates	reference^||^
**QALYs**				
*Unadjusted QALY*	0.787 (0.074)	0.782 (0.062)	0.800 (0.067)	0.800 (0.064)
SF-6D				
*Adjusted QALY*^ý^ SF-6D	0.787 (0.061)	0.783 (0.054)	0.800 (0.059)	0.800 (0.056)
*Adjusted Incremental QALY* ^ý^	0.004(0.001)	reference^||^	-0.001 (0.002)	reference^||^
**Cost-utility analysis** ^Ý^				
*Incremental cost per QALY*	5946 (95% CI: -50075 to 30986) Upper right quadrant	reference^||^	238913 (95% CI: 105355 to 772945) Lower left quadrant	reference^||^

Notes: The time horizon for this analysis was 12-months.

* p<0.05

Ý ICER based on total health resource utilization costs and cost of delivering programs

ý Incremental QALYs are adjusted for the baseline utility using a linear regression model

|| Reference indicates that the comparator is the usual care (control) group.

#### Adjusting QALYs for baseline utility

For women, the incremental adjusted QALYs after 12 months and adjusted for baseline SF-6D, was 0.001 (SD: 0.008) for the complete case and 0.004 (SD: 0.001) for the imputed cases for OEP compared with usual care, For men, the incremental adjusted QALYs after 12 months and adjusted for baseline SF-6D, was 0.005 (SD: 0.010) for the complete case and -0.001 (SD: 0.002) for the imputed case.

#### Complete & imputed case analysis

**Fig 1:**
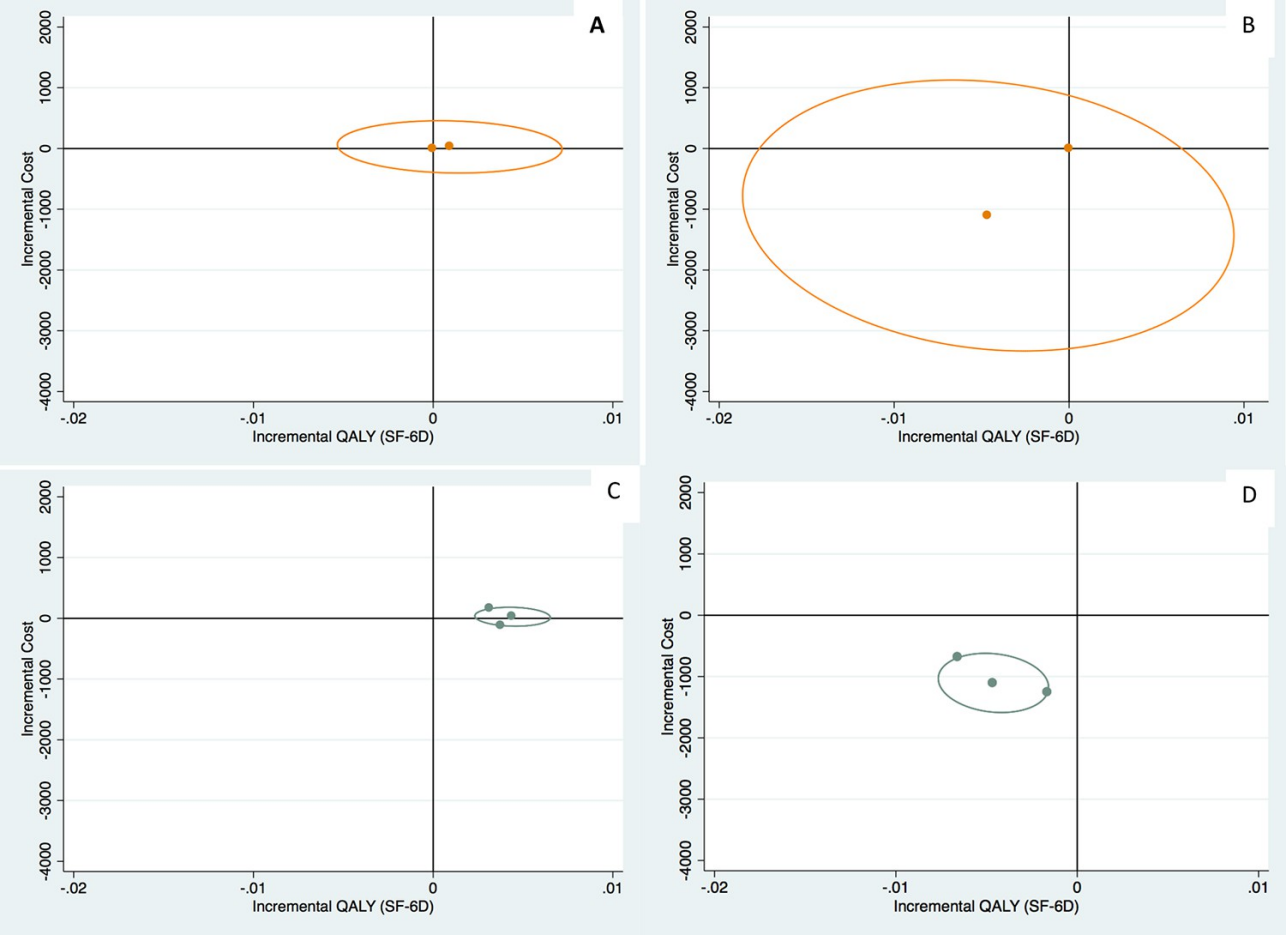
a: Cost-effectiveness plane: Complete case analysis for women, incremental cost-utility ratio based on QALYs estimated from the SF-6D. b: Cost-effectiveness plane: Complete case analysis for men, incremental cost-utility ratio based on QALYs estimated from the SF-6D. c: Cost-effectiveness plane: Imputed case analysis for women, incremental cost-utility ratio based on QALYs estimated from the SF-6D. d: Cost-effectiveness plane: Imputed case analysis for men, incremental cost-utility ratio based on QALYs estimated from the SF-6D.

For women, the cost-utility analyses demonstrated that for OEP, compared with usual care, the bootstrapped cycles were represented in the north and south quadrant intersecting the east and west quadrant with approximately equal proportions for the complete case analysis; and 100% in the east quadrant for the imputed analyses (Tables 2 and 3, Fig 1A and 1C). For men, the cost-utility analyses demonstrated that for OEP, compared with usual care, 100% of the bootstrapped cycles were represented in the south-west quadrant (i.e., cost-saving region) for the imputed case analysis. (Tables 2 and 3, Fig 1B and 1D). The results of the complete case analysis for men were comparable with the majority of the bootstrapped cycles in the south-west quadrant with some east quadrant representation of the incremental QALYs. The incremental QALYs were close to zero and the OEP did not result in substantive changes to quality of life. The OEP did result in cost-savings for men from a health care system perspective.

## Discussion

The efficacy of OEP as a secondary falls prevention strategy did not vary by gender. Our study indicates that among older men who have had a fall, the OEP is a cost-saving secondary prevention strategy. Among men, the OEP saved health resource utilization costs while significantly preventing falls. In contrast, among women, the OEP did not save healthcare costs and lead to a non-significant reduction in the IRR for falls compared with usual care. For men and women, the incremental QALY gains or losses were not clinically significant, thus resulting in no meaningful changes in health-related quality of life. The directionality of these results suggests that gender may be an important factor to consider when ascertaining the efficacy and the cost-effectiveness of the OEP.

The efficacy of the OEP did not vary as function of gender, which aligns with past OEP research. A meta-analysis that included four randomized controlled trials concluded that the OEP was efficacious for women and men. Women had a 26% significant reduction in falls rate and men had a 60% significant reduction in falls rate [19]. Similar to our study, men had a higher rate of falls reduction.

Our stratified analyses of women and men showed more benefits for men. It is likely men were frailer than women. The condition frailty is a physiological condition that demonstrates differences by gender [47]. Gender related effects may result in differential intervention efficacy for women and men. Prior research supports the notion that the OEP is most efficacious and cost-effective among those at highest risk (e.g., men may be more frail) [48]. Hence, it is conceivable that some interventions may have differential impacts based on individuals baseline frailty. As such this may be one plausible reason for observing differences by gender.

The gender differences in our stratified analyses may be explained by gender differences in health seeking behaviours. One study examining health resource utilization of older adults living in Spain observed a higher percentage of women seeking preventative or diagnostic health care services or resources [49]. Men made greater use of emergency services. These differences may be due to: 1) women may be more proactive in seeking care earlier, 2) the number and type of fall risk factors may differ by gender [50] (e.g., number of chronic diseases may be higher in women [51]), and 3) men may be more likely to engage in risk-taking behaviour and overestimate their true ability [52], and 4) baseline health related quality of life may differ [53]. In our study, men also demonstrated higher mean health resource utilization costs. It is plausible the increased health resource utilization costs were observed because men may be more frail and thus accessing care they require.

These findings have potential implications. Relevant to clinical management of older adults who fall, frailty may be an important biological factor and health seeking behavior may be a key social factor to examine. Men may have lower health seeking behavior and thus present further along the frailty spectrum [54]. The OEP is most cost-effective amongst those at highest risk of future falls. Hence, if men are frailer than women at the onset of the OEP, it may explain the observed greater benefit of the OEP among men. As such, these findings may assist in guiding future resource allocation decisions. Future research is needed to explore whether frailty, among women and men, may be a useful indicator of determining the cost-effectiveness of the OEP.

This study has limitations. This study was not powered apriori to examine differences by gender. Stratified analyses are subject to bias. Men in the OEP group had a higher baseline mean number of falls and thus may be frailer at baseline. Costs were collected using the health resource utilization questionnaire, which may be subject to recall bias. Participants used a monthly diary to track and report their health care resource utilization to minimize the anticipated recall bias. Falls were collected prospectively via monthly falls diaries. Because we expected comparable levels of recall bias between groups, we did not estimate any impact on the incremental cost-effectiveness/utility ratio.

## Conclusion

We did not observe a gender by intervention interaction effect for falls IRR. The best value for money of the OEP was among men where the OEP was cost-saving. It is conceivable that men were frailer at baseline. The OEP did not change the health-related quality of life of older fallers among women and men. These findings suggest that future research should explore variables related to gender (i.e., health seeking behaviours, frailty) differences in order to understand the underlying reasons for observed differences among women and men.

## Supporting information

S1 Checklist CONSORT 2010 checklist. (DOC)
